# Whole Transcriptome Sequencing Enables Discovery and Analysis of Viruses in Archived Primary Central Nervous System Lymphomas

**DOI:** 10.1371/journal.pone.0073956

**Published:** 2013-09-04

**Authors:** Christopher DeBoever, Erin G. Reid, Erin N. Smith, Xiaoyun Wang, Wilmar Dumaop, Olivier Harismendy, Dennis Carson, Douglas Richman, Eliezer Masliah, Kelly A. Frazer

**Affiliations:** 1 Moores Cancer Center, University of California San Diego, La Jolla, California, United States of America; 2 Bioinformatics and Systems Biology Graduate Program, University of California San Diego, La Jolla, California, United States of America; 3 Department of Pediatrics and Rady Children’s Hospital, University of California San Diego, La Jolla, California, United States of America; 4 Department of Pathology, University of California San Diego, La Jolla, California, United States of America; 5 VA San Diego Healthcare System and Center for AIDS Research, University of California San Diego, La Jolla, California, United States of America; 6 Department of Neurosciences, University of California San Diego, La Jolla, California, United States of America; 7 Clinical and Translational Research Institute, University of California San Diego, La Jolla, California, United States of America; 8 Institute for Genomic Medicine, University of California San Diego, La Jolla, California, United States of America; University of Southern California Keck School of Medicine, United States of America

## Abstract

Primary central nervous system lymphomas (PCNSL) have a dramatically increased prevalence among persons living with AIDS and are known to be associated with human Epstein Barr virus (EBV) infection. Previous work suggests that in some cases, co-infection with other viruses may be important for PCNSL pathogenesis. Viral transcription in tumor samples can be measured using next generation transcriptome sequencing. We demonstrate the ability of transcriptome sequencing to identify viruses, characterize viral expression, and identify viral variants by sequencing four archived AIDS-related PCNSL tissue samples and analyzing raw sequencing reads. EBV was detected in all four PCNSL samples and cytomegalovirus (CMV), JC polyomavirus (JCV), and HIV were also discovered, consistent with clinical diagnoses. CMV was found to express three long non-coding RNAs recently reported as expressed during active infection. Single nucleotide variants were observed in each of the viruses observed and three indels were found in CMV. No viruses were found in several control tumor types including 32 diffuse large B-cell lymphoma samples. This study demonstrates the ability of next generation transcriptome sequencing to accurately identify viruses, including DNA viruses, in solid human cancer tissue samples.

## Introduction

Non-Hodkin’s lymphomas are tied with Kaposi sarcoma as the most common AIDS-defining cancers. Primary central nervous system lymphomas (PCNSL) accounted for 7% of all AIDS cancers in the early HAART era and have a roughly 1000-fold increased prevalence in persons living with AIDS [1], [2]. While the incidence rate of PCNSL has fallen by nearly 90% since the advent of HAART, immunocompromised individuals continue to be at risk for this aggressive cancer [3]. A strong association has been established between PCNSL and Epstein Barr virus (EBV, human herpesvirus 4, HHV4), a 170 kb double stranded DNA virus associated with infectious mononucleosis as well as numerous human malignancies [4], [5], [6], [7], [8]. EBV efficiently immortalizes B-cells *in vitro* and is associated with cancers besides PCNSL such as Burkitt’s and Hodgkin lymphomas, nasopharyngeal carcinoma, and others [8], [9], [10]. It has been hypothesized that viruses in addition to EBV may play a role in PCNSL [11], [12].

Next generation transcriptome sequencing offers the ability to detect viruses with few *a priori* assumptions regarding gene sequences or a sample’s viral population. Previous experimental approaches for identifying viruses in host samples relied on PCR or microarrays to identify viral sequences. PCR-based approaches are confounded by the need to clone viruses or design primers for genomes that can be highly polymorphic or poorly characterized [13], [14]. Similarly, microarray-based expression studies cannot characterize the viral population of a sample without using probes specific to viral sequences, limiting the search for unexpected or highly polymorphic viruses [15]. In contrast, high throughput sequencing allows for a more unbiased and complete view of the viral population in a sample. Previous high throughput studies removed sequencing reads that aligned to the reference human genome or transcriptome and mapped remaining reads against a viral database [16], [17]. Such computational subtraction methods have been used to study melanoma and squamous cell conjunctival carcinoma and led to the identification of the Merkel cell polyomavirus that causes Merkel cell carcinoma, an aggressive skin cancer [18], [19], [20], [21]. More recent methods that do not rely on subtracting host-derived sequences have identified viruses in sweet potato and correctly identified pathogens in HIV-infected cells and the transformation virus in a prostate cancer cell line [22], [23], [24].

Here we have performed highly parallel transcriptome sequencing of four AIDS-related PCNSL tissue samples and built upon previous analysis methods to identify expected and unexpected viruses and characterize viral gene expression. We were able to identify EBV in all four PCNSL samples, consistent with previous studies that have reported finding EBV in effectively all AIDS-related PCNSL patients [4], [25], [26], [27], as well as unexpected viruses in one of the samples. This is among the first studies to use next-generation sequencing methods to identify unexpected viruses in human cancer tissue samples and provides the framework for performing this type of analysis in larger cancer datasets such as those being generated by The Cancer Genome Atlas and other consortia [28], [29], [30].

## Materials and Methods

### Samples for which Transcriptomes were Generated by SOLiD Sequencing

#### PCNSL brain specimens

1 mm bores were extracted from HIV-positive PCNSL post-mortem brain tumor samples from two 37 year old non-Hispanic white cases (PCNSL4 and PCNSL2) and two Hispanic cases, aged 37 and 41 (PCNSL1 and PCNSL3 respectively), from the California NeuroAIDS Tissue Network (CNTN) [31]. All patients were diagnosed with PCNSL. Additionally, PCNSL3 was diagnosed with progressive multifocal leukoencephalopathy (PML), microglial nodular encephalitis, and cytomegalovirus ventriculitis. PCNSL2 was diagnosed with microglial nodule encephalitis and CE 114 was diagnosed with microglial nodular encephalitis in the medulla and pons consistent with CMV encephalitis, B-cell lymphoma in the temporal cortex, leukoencephalopathy of anterior commisure, and infarction of the occipital cortex (Table 1).

**Table 1:** Clinical diagnoses besides PCNSL for four PCNSL patients.

Subject	Additional diagnoses	Associated infection
PCNSL1	Aspergillosis	*Aspergillus*
PCNSL1	Hepatitis B	Hepatitis B virus
PCNSL1	Hepatitis C	Hepatitis C virus
PCNSL2	Microglial nodule encephalitis	CMV [60]
PCNSL3	Progressive multifocalleukoencephalopathy	JCV [47]
PCNSL3	Microglial nodular encephalitis	CMV
PCNSL3	Cytomegalovirus ventriculitis	CMV [60]
PCNSL4	Microglial nodular encephalitis	CMV
PCNSL4	Leukoencephalopathy ofanterior commissure	
PCNSL4	Infarction of the occipitalcortex	

#### Transduced cord blood

To serve as a positive control a cord blood sample was purchased from AllCells (Emeryville, CA). Peripheral blood mononuclear cells (PBMC) were extracted from peripheral blood following Ficoll density centrifugation and CD34^+^ cells were then purified by magnetic bead separation (MACS; Miltenyi, Bergisch Gladbach, Germany). These cells were transduced with lentiviral vectors, FACS sorted using human-specific CD34 and CD38 antibodies and a lineage cocktail directly into RLT buffer (Qiagen, Valencia, CA) as described previously [32].

#### Non-small Cell Lung Carcinoma (NSCLC)

Resected tumor from a 75-year-old man with a 30-pack-year history of smoking and an incidentally found squamous cell carcinoma was xenografted into NSG mice and harvested after palpable tumor growth was observed as previously described [33]. Tumor cells were sorted into CD133−/EpCAM+ and CD133+/EpCAM+ populations. Both subpopulations were examined.

#### Chronic Lymphocytic Leukemia (CLL)

Two CLL samples were obtained from the CLL Consortium. Sample CLL1 is from a Caucasian female age 70 with indolent disease while CLL2 is from a Caucasian male age 63 with aggressive disease. CLL1 was found to have chr13 deletion in 72% of cells and CLL2 had trisomy 12 in 66% of cells according to FISH.

### SOLiD Library Preparation and Sequencing

RNA was isolated from samples with the QIAGEN RNeasy kit and analyzed on the Bioanalyzer (Agilent, Santa Clara, CA, USA). Whole transcriptome libraries were prepared following “SOLiD Total RNA-Seq kit protocol” (Life Technologies, Carlsbad CA, USA). Total RNA samples were depleted using “RiboMinus Eukaryote Kit for RNA-Seq” (Invitrogen, Carlsbad CA, USA). One PCNSL total RNA sample (PCNSL3) was non-depleted due to low amount of starting material (∼1.2 ug). The total RNA or rRNA-depleted total RNA were fragmented by RNAse III and cleaned up using RiboMinus concentration Module (Invitrogen, Carlsbad CA, USA). RNA fragments were hybridized and ligated to SOLiD adaptor mix and reverse transcription performed. Purified cDNA (Qiagen, Valencia, CA, MinElute PCR purification kit) was amplified 15 cycles using SOLiD 3′ and SOLID 5′ primers. Amplified DNA were purified using PureLink PCR Micro kit (Invitrogen, Carlsbad CA, USA), QC by Bioanalyzer 2100 DNA 1000 kit (Agilent, Santa Clara, CA, USA), and quantified by Qubit (Invitrogen, Carlsbad CA, USA). The whole transcriptome libraries were used for making SOLiD templated beads following SOLiD4 System Templated Bead Preparation Guide. 50 bp fragment sequencing was performed on the ABI SOLiD3 platform generating between 472 and 525 million raw reads per sample for PCNSL samples and similar amounts for other samples.

### Samples for which Transcriptomes were Generated by Illumina GAII

#### Diffuse large B-cell lymphoma

32 diffuse large B-cell lymphoma transcriptomes were sequenced on the Illumina GAII as part of the Cancer Genome Characterization Initiative (http://cgap.nci.nih.gov/cgci.html) [GenBank:phs000235]. Sequencing methods and information are available through the Short Read Archive.

### Analysis Pipeline, Alignment and Virus Detection

#### Alignment to viral database

Raw sequencing reads were converted from csfasta to csfastq files using solid2fastq (-b, default options otherwise) and aligned against a viral database consisting of 3,906 complete viral genomes (ftp://ftp.ncbi.nih.gov/refseq/release/viral/) using BWA (-c, -l 25) to select for reads that were likely of viral origin and aligned reads were sorted with SAMtools [34], [35].

#### Viral read filtering

Low complexity reads, polysequences, and reads that aligned to either a vector database (ftp://ftp.ncbi.nih.gov/pub/UniVec/) or a human database consisting of the hg19 genome plus RefSeq, Ensembl, and UCSC genes were removed using SeqClean (-l 30, 30 bp overlap for BLAST search, default options otherwise, http://compbio.dfci.harvard.edu/tgi/software/) to enrich for informative reads.

#### Read assembly and alignment to nucleotide database

Viral reads that remained after filtering were assembled using Velvet (hash length 21, default options otherwise) to provide contigs that would align to viruses uniquely and with high significance. These contigs were aligned against the NCBI nucleotide (nt) sequence database using blastn (default parameters) [36].

#### Analyzing BLAST results for significance

The blastn matches were processed using the analysis script from Isakov *et al.* which calculates *E*-values that indicate which viruses and strains are likely present [23]. Briefly, blastn matches with the lowest *E*-value and highest alignment score on a per sample basis were used to calculate a virus table for each sample. This table reported for each virus a total *E*-value that was calculated by multiplying the *E*-values of all unique matches for that virus (*E*-values are comparable to *p*-values for *E*<0.01). To aid in intraspecies differentiation, a total score for each virus was calculated by summing the blastn scores of unique matches with the scores of multiple matches (matches that align equal well to multiple viruses) divided by the different number of viruses the hit aligns to [23].

#### Realignment and variant calling

After identifying viruses present in each sample, the viral reads were realigned using BWA to a reference consisting of only EBV, JCV, CMV, and HIV for visualization, variant calling, and expression estimation. For variant calling, duplicate reads were removed using Picard tools and pileup files were generated from reads with mapping quality greater than 30 using Samtools. Variants were considered significant if the total coverage of the site and the variant was greater than five and the variant allele was present at a frequency greater than 10%. Read coverage for EBV genes was calculated by intersecting the realigned reads with the NCBI viral gene annotation (ftp://ftp.ncbi.nih.gov/refseq/release/viral/) and counting the number of reads that overlapped each gene (intersectBed -c) [37].

### Availability of Supporting Data

The data set supporting the results of this article is available in the Short Read Archive repository [SRP017364, http://www.ncbi.nlm.nih.gov/Traces/sra/sra.cgi?study=SRP017364].

### Ethics Statement

This research was submitted to the University of California San Diego Human Research Protections Program and was found to be exempt since the tissue is post-mortem. The CLL patient was consented by the CLL Research Consortium (http://cll.ucsd.edu/) under approval by the University of California San Diego Human Research Protections Program. The NSCLC patient provided written informed consent following UCSD Institutional Review Board approved protocols and the mouse experimental procedures were approved by the Pfizer Global Research and Development Institutional Animal Care and Use Committee as previously described [33].

## Results and Discussion

### Transcriptome Analysis of Cancer Specimens for Viral Sequences

To identify additional viruses besides EBV that may play a role in PCNSL we performed high throughput transcriptome sequencing on four frozen post-mortem brain tumor samples from HIV-positive PCNSL patients with clinical diagnoses available from the California NeuroAIDS Tissue Network (CNTN). Samples were obtained from two 37-year-old non-Hispanic white patients, PCNSL2 and PCNSL4, and two Hispanic patients, PCNSL1 and PCNSL3, aged 37 and 41 respectively [SRA:SRP017364]. The four patients had clinical diagnoses consistent with heavy viral load including hepatitis, microglial nodule encephalitis, and progressive multifocal leukoencephalopathy in addition to PCNSL (Table 1). An average of 492,914,707 raw reads (standard deviation 23,205,410) were generated for each PCNSL sample. As described in further detail above, the sequencing reads were assembled, BLASTed against the NCBI nucleotide database and total *E*-scores calculated for each virus in each sample to identify viruses present (Table 2). No viruses were found in chronic lymphocytic leukemia (CLL) or non-small cell lung carcinoma (NSCLC) negative controls, but HIV 1 and synthetic construct were found as expected in one transduced cord blood sample which served as a positive control [SRA:SRP017364]. No viruses were identified in a set of 32 diffuse large B-cell lymphoma (DLBCL) samples downloaded from the Cancer Genome Characterization Initiative (Table S1). Notably, none of these samples are EBV infected which is consistent with the low incidence of EBV infection for diffuse large B-cell lymphoma (see Supplementary Materials) [38].

**Table 2:** Viruses found in each sample and corresponding total *E*-value and total BLAST score.

Subject	Cancer Type	Virus Identified	Total *E*-value	BLAST score	Number of reads*
PCNSL1	AIDS-related PCNSL	EBV	<1e-200	1785	10492
PCNSL2	AIDS-related PCNSL	EBV	<1e-200	27485	57298
PCNSL3	AIDS-related PCNSL	EBV	<1e-200	647	901
		JCV	<1e-200	4901	19430
		CMV	<1e-200	7145	4114
PCNSL4	AIDS-related PCNSL	EBV	1.21e-57	145	37
		HIV	<1e-200	2384	1283
CORD1	Experimentally transducedcord blood	HIV	<1e-200	2229	2644
		Synthetic construct	1.32e-162	1900	NA**
NSCLC1	NSCLC (CD133-)	None			
NSCLC2	NSCLC (CD133+)	None			
CLL1	CLL (indolent disease)	None			
CLL2	CLL (aggressive disease)	None			

* Number of reads mapped to the whole genome following realignment to only EBV, JCV, CMV, and HIV.

** This synthetic construct was not in the NCBI viral database, so it was not used for realignment.

### EBV

**Figure 1:**
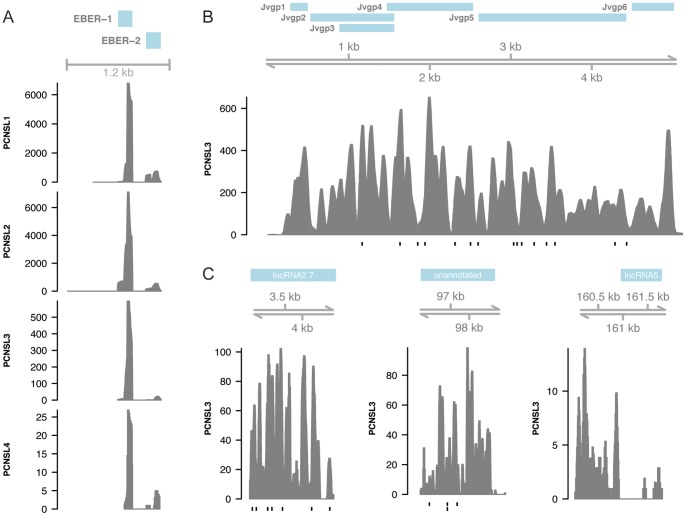
Viral read coverage. A. Read coverage (number of reads) across EBV latent genes EBER-1 and EBER-2 in four PCNSL samples. B. Read coverage across the entire JCV genome in PCNSL3. C. Read coverage across three cytomegalovirus ncRNA in PCNSL3. All alignments produced using BWA (25 bp seed, default options otherwise). Vertical black lines below histograms indicate variants relative to the reference sequence.

We identified EBV expression in all four PCNSL samples by identifying reads that mapped to Epstein-Barr encoded RNA (EBER) 1 and EBER 2 (Table 2, Figure 1A). The total BLAST score (as calculated according to Isakov *et al.*) for EBV varied between samples, reflecting differing numbers of reads mapping to the virus in each sample. The discovery of the EBER non-coding RNA’s (ncRNA) in all four PCNSL samples serves as a positive control for the viral discovery method used here since EBV is known to be associated with PCNSL and the EBER ncRNAs are known to be transcribed in large quantities (∼10^7^ transcripts per cell) in EBV-transformed cells during latent infection [39]. Interestingly, the sample from PCNSL2 had roughly 50 times more reads mapped uniquely across most of the EBV genome compared to the other samples (Table 2). The samples from PCNSL1 and PCNSL2 demonstrated similarly high expression levels of the EBER ncRNA’s compared with the samples from the other two subjects, even though fewer reads mapped across the EBV genome in PCNSL1 (Table 3).

**Table 3:** Read coverage over select EBV genes for PCNSL samples.

			Sample coverage			
Gene	NCBI ID	Coordinates	PCNSL1	PCNSL2	PCNSL3	PCNSL4
BALF4	HHV4tp2_gp73	157775–160348	151	373	2	0
BHRF1	HHV4tp2_gp07	42204–42779	18	10355	16	0
EBER-1	HHV4tp2_gs01	6634–6800	7693	8724	727	27
EBER-2	HHV4tp2_gs02	6961–7133	1571	1047	30	6
LMP-1	HHV4tp2_gp80	169188–170457	3	1362	0	0
LMP2A	HHV4tp2_gp01	1–1680	27	134	1	0
LMP2A	HHV4tp2_gp01	167587–172764	13	3118	0	0
Genome		1–172764	10492	57928	901	37

EBV is known to express 10 genes during lymphoproliferation and at least three genes during latent infection, including EBER 1, EBER 2, and latent membrane protein 2A (LMP2A) [40]. LMP2A expression was observed in the samples from patients PCNSL1 and PCNSL2 and based on the number of mapping reads LMP2A transcripts were present at a lower frequency (∼175 times) than the EBER1 transcripts in these samples. For instance, in the sample from PCNSL2 after realigning all viral reads to a database consisting of only HIV, JCV, CMV, and EBV, 8,724 reads mapped to EBER 1 and 134 reads mapped to LMP2A (Table 3). LMP2A is known to provide survival signals to B-cells and can constitutively activate the Ras/PI3K/Akt pathway leading to B-cell survival and resistance to apoptosis, suggesting a possible mechanism of tumorigenesis in samples with LMP2A expression [41], [42]. LMP2A is also known to be expressed during EBV latent infection and may not be active at this advanced stage of PCNSL [7]. The lack of reads mapping to LMP2A in the samples from patients PCNSL3 and PCNSL4 does not necessarily indicate that the transcript is not present but may instead be a result of overall lower coverage of EBV. Overall, these data suggest that patients PCNSL2 and PCNSL1 may have had more active EBV infections and/or higher quality tissue samples than the other two subjects.

The relatively high read coverage across the EBV genome in PCNSL2 allowed us to examine the sequences for single nucleotide variants (SNV’s) relative to the EBV reference. We identified 37 SNVs, of which 24 were in protein coding genes and 13 were located in intergenic regions. There are a similar number of putative variants in the PCNSL sample from PCNSL1, but these variants were not deemed significant due to the lower read coverage across the EBV genome. There are two EBV types present in the NCBI viral database used here; the BLAST scores indicate that the viruses in each sample are more similar to EBV type 2 (NC_009334.1) because more contigs map uniquely to this reference sequence. This categorization may not be accurate, however, because EBV types 1 and 2 are normally distinguished by investigating the sequence of the EBNA-2 and EBNA-3 genes which was not possible here due to the lack of read coverage for these genes. While an individual can be infected with multiple EBV strains or both subtypes, PCNSL2 is likely infected with a single strain because the 37 variants observed have 100% alternate allele frequency [43], [44]. There are known to be EBV strains within each subtype but these strains are not cataloged in the NCBI viral database, so it is difficult to determine whether this strain has been identified previously.

### JC Polyomavirus

We identified JC polyomavirus (JCV) in the sample from PCNSL3 by BLASTing contigs assembled from the sequencing reads against the viral database. Investigating the alignment of the reads to the viral database showed reads covering nearly the entire JCV genome (Figure 1B) [45]. This is consistent with previous reports that describe “giant” RNAs larger than the viral genome that are transcribed after JCV DNA replication has begun [46]. The discovery of JCV in PCNSL3 is consistent with this patient’s diagnosis with progressive multifocal leukoencephalopathy (PML), a demyelinating disease resulting in destruction of oligodendrocytes caused by an active infection of the virus (Table 1) [47]. JCV has also been shown to infect and replicate in B-lymphocytes [48], [49], [50]; thus it is unclear whether our finding is due to an active virus infection of the cancerous B-cells, the adjacent oligodendrocytes, or both. The immune-related pathology of PCNSL and the oncogenic potential of JCV have led some to hypothesize an association between JCV and PCNSL [51], [52]. The findings of previous studies conflict however, with some indicating that JCV is often found in PCNSL samples and others indicating that the virus is not found in PCNSL samples [11], [53], [54]. The results presented here do not have the power to definitely support either hypothesis but indicate that further work may be needed to understand whether JCV plays a role in PCNSL.

The other three patients (PCNSL1, PCNSL2 and PCNSL4) were not diagnosed with PML, so we did not expect to find evidence of an active JCV infection. Interestingly, PCNSL1’s sample did have 18 unique reads that mapped to JCV and the samples from PCNSL4 and PCNSL2 had four and one reads that mapped to JCV, respectively. However, none of the samples besides that of PCNSL3 reached significance for JCV (*E*<0.01) after Velvet assembly and BLASTing against the nucleotide database.

In the PCNSL sample from patient PCNSL3 15 SNVs were identified relative to the JCV reference sequence, of which 13 are in protein coding genes while the remaining two are in intergenic regions. The alternate allele frequency was 100% for all but two of the SNV’s. These two variants may represent novel mutational events in this individual since mutations have been observed in JCV DNA sequence among parent-children pairs, indicating that JCV can acquire mutations in the span of an individual infection [55]. Interestingly, of the 18 reads that mapped to JCV in PCNSL1’s sample, three reads differed from the reference at one base; these three variants agree with those observed in PCNSL3’s sample. This finding suggests a low-grade JCV infection may have been present in a nearby site in this patient that did not reach clinical significance and also underscores the need for more dynamic reference sequences that take into account known viral polymorphisms and variation. Currently, the NCBI viral database has only one reference sequence for most viruses, but as shown here, viral sequence variation can be quite high.

### Cytomegalovirus (CMV)

CMV was identified in PCNSL3’s sample consistent with this patient’s diagnosis with microglial nodular encephalitis and cytomegalovirus ventriculitis (Tables 1 and 2). CMV is a double-stranded DNA virus with the largest genome among human herpesviruses at 235 kb [56]. Investigating the viral read alignments visually allowed for the identification of expressed viral genes [45]. Nearly all of the reads aligning to CMV mapped to two long noncoding RNA’s (lncRNA’s), lncRNA2.7 and lncRNA5, as well as an unannotated RNA (Figure 1C). The unannotated RNA contains several open reading frames but they do not correspond to the read alignments observed at this locus leading us to suspect that this is another lncRNA. These three lncRNA’s were recently found to be highly expressed during CMV infection [57]. lncRNA2.7 has been reported to prevent apoptosis while the homolog of lncRNA5 in mice has been found to promote virulence [58], [59]. The role of the unannotated lncRNA is unknown. We did not observe reads mapping to the CMV microRNA loci as reported elsewhere [57], but this may be due to the library preparation method, which would preclude identification of such short transcripts.

CMV was not observed in the samples from either PCNSL4 or PCNSL2 despite clinical evidence for CMV infection (Table 1). This is likely due to a lack of CMV-infected cells in the tumor samples or the low rate of inclusion in CMV infection [60]. CMV has not been hypothesized to play a role in PCNSL and likely does not contribute to the disease in PCNSL3. However, it is interesting to note that cancerous B-cells coinfected with EBV and CMV could utilize the anti-apoptotic properties of CMV lncRNA2.7 to avoid cell death.

Comparison of sample PCNSL3 aligned reads to the CMV reference genome identified eight SNV’s, two two-base-pair deletions, and one two-base-pair insertion. Several more putative SNV’s and indels were identified but did not reach significance. Six of the SNV’s and one deletion are located in lncRNA2.7 and the other SNV and indels are located in the newly identified ncRNA. The insertion and deletion identified in this ncRNA are both located at the beginning of an AT repeat. The alternate allele frequencies of the variants ranged from 14% to 100% which may indicate infection with multiple strains of CMV, a common occurrence in immunocompromised individuals [61]. A number of genes have been found to be highly variable in CMV, but the mutated genes here do not overlap with that list [56].

### HIV

HIV was discovered in sample PCNSL4 with reads mapping sparsely throughout the HIV genome. The low read coverage for this virus precluded gene expression analysis, but 14 SNV’s relative to the reference sequence were identified. Ten of these variants are in *pol,* two are in *env*, and there is one each in *tat* and *gag.* Many more sites contained alternate base calls but did not reach significance due to the low total read coverage at these sites. These alternate alleles may represent different HIV strains in the patient, but a deeper sequencing depth is needed to confirm this. The presence of HIV indicates possible contamination of this tumor sample with non-B-cells such as glial cells, which can be infected with HIV [62]. This sample also had the lowest total BLAST score for EBV due to the relatively small number of reads mapping to EBV which is consistent with low tumor purity.

## Conclusions

We have effectively demonstrated the use of RNA-seq for identifying and characterizing viruses in frozen solid tumor samples. EBV was identified in the four PCNSL archived samples; additionally, JCV and CMV were found in one sample and HIV was found in one sample. We were able to identify two known and one unannotated lncRNA in CMV that have recently been reported as highly transcribed during active infection [57]. Given that lncRNA2.7 and lncRNA5 influence apoptosis and virulence, this unannotated lncRNA may play an important role in CMV pathogenesis. Further characterization of CMV gene expression over the course of an infection may enhance our understanding of this unannotated lncRNA and studies should be performed to test for function *in vivo*. We also identified variants and indels in EBV, HIV, CMV, and JCV. The diversity of the viral sequences observed here relative to the reference sequences underscore the need for expanded references that incorporate different viral strains and known polymorphisms. Such references might be constructed in a semi-automated fashion by mining the rapidly growing amount of archived sequencing data for common variation. The methods described herein can readily be applied to any transcriptome data and will generalize to identifying bacteria or any other sequences of interest. Such approaches offer the opportunity to investigate pathogenic infection without any prior assumptions regarding expected pathogens.

## Supporting Information

Table S1 Viruses found in 32 large diffuse B-cell lymphoma samples and corresponding total *E*-value and total score.
(DOCX)
