# Supplementary material for: Whole Transcriptome Sequencing Enables Discovery and Analysis of Viruses in Archived Primary Central Nervous System Lymphomas
Source: PLoS One. 2013 Sep 4;8(9):e73956. doi: 10.1371/journal.pone.0073956 (PMC3762708; doi:10.1371/journal.pone.0073956)
Supplement: Table S1 — Viruses found in 32 large diffuse B-cell lymphoma samples and corresponding total E-value and total score. (DOCX) [file pone.0073956.s001.docx]

phiX174 and several acinetobacter phage were found to be significant in a subset of 32 diffuse large B-cell lymphoma samples downloaded from the Cancer Genome Characterization Initiative (Supplementary Table 1). Pseudomonas phage LKA1 was found in one sample and another sample unexpectedly contained two lactococcus bacterial strains and several lactococcus phage strains. The phiX174 present in the samples is consistent with the phage’s use as a sequencing control, but the origin of the other viral matches is unclear. They may represent infection, laboratory contamination, or false positives from our detection pipeline. Visual inspection of the read alignments for these samples shows that the reads do not typically pileup but rather align somewhat randomly across the genomes of the significant viruses and bacteria. This likely indicates laboratory contamination from vectors or some other source of DNA. Importantly, none of these samples are EBV infected which is consistent with the low incidence of EBV infection for diffuse large B-cell lymphoma.

Table S1. Viruses found in 32 large diffuse B-cell lymphoma samples and corresponding total *E*-value and total score.

| Sample | Virus Identified | Total *E*-value | Total score |
| --- | --- | --- | --- |
| SRS010533 | Pseudomonas phage LKA1 | 5.40E-51 | 133 |
| SRS010534 |  |  |  |
| SRS010535 | Enterobacteria phage S13 | 6.00E-68 | 143 |
|  | Enterobacteria phage phiX174 | $<{10}^{-200}$ | 2379 |
| SRS010536 |  |  |  |
| SRS010537 | Enterobacteria phage phiX174 | $<{10}^{-200}$ | 4030 |
| SRS010538 | Enterobacteria phage phiX174 | $<{10}^{-200}$ | 3704 |
| SRS010539 | Enterobacteria phage S13 | $<{10}^{-200}$ | 385 |
|  | Enterobacteria phage phiX174 | $<{10}^{-200}$ | 431 |
|  | Acinetobacter phage 133 | 1.25E-54 | 119 |
| SRS010540 | Enterobacteria phage phiX174 | $<{10}^{-200}$ | 4151 |
| SRS010541 | Enterobacteria phage S13 | 1.75E-161 | 312 |
|  | Enterobacteria phage phiX174 | $<{10}^{-200}$ | 2894 |
| SRS010542 | Enterobacteria phage phiX174 | 3.19E-212 | 530 |
| SRS010543 |  |  |  |
| SRS010544 |  |  |  |
| SRS010545 |  |  |  |
| SRS010546 | Enterobacteria phage phiX174 | $<{10}^{-200}$ | 3274 |
| SRS010547 | Enterobacteria phage S13 | $<{10}^{-200}$ | 733 |
|  | Enterobacteria phage phiX174 | $<{10}^{-200}$ | 4015 |
|  | Acinetobacter phage Acj61 | 6.01E-63 | 155 |
| SRS010548 | Enterobacteria phage phiX174 | $<{10}^{-200}$ | 1889 |
| SRS010549 | Enterobacteria phage phiX174 | $<{10}^{-200}$ | 1215 |
| SRS010550 | Acinetobacter phage Acj61 | 3.18E-120 | 281 |
| SRS010551 | Acinetobacter phage Acj61 | 7.72E-137 | 333 |
| SRS010552 |  |  |  |
| SRS010553 | Acinetobacter phage 133 | 9.29E-62 | 132 |
|  | Enterobacteria phage phiX174 | $<{10}^{-200}$ | 1452 |
| SRS010554 |  |  |  |
| SRS010555 |  |  |  |
| SRS010556 |  |  |  |
| SRS010557 |  |  |  |
| SRS010558 |  |  |  |
| SRS010559 | Acinetobacter phage Acj61 | 8.58E-73 | 195 |
| SRS010560 | Enterobacteria phage phiX174 | 1.36E-74 | 176 |
| SRS010561 | Lactococcus phage phiLC3 | 3.48E-60 | 129 |
|  | Enterobacteria phage phiX174 | 6.54E-57 | 123 |
|  | Lactococcus phage r1t | 1.72E-62 | 154 |
|  | Lactococcus lactis subsp. lactis KF147 | 1.09E-53 | 138 |
|  | Lactococcus phage 4268 | $<{10}^{-200}$ | 1194 |
|  | Acinetobacter phage Acj61 | 1.33E-94 | 234 |
|  | Lactococcus lactis subsp. lactis CV56 | 2.73E-135 | 328 |
| SRS010562 | Acinetobacter phage 133 | 9.08E-62 | 132 |
| SRS010563 |  |  |  |
| SRS011785 |  |  |  |
